# Complete Genome Sequence of Halorubrum ezzemoulense Strain Fb21

**DOI:** 10.1128/MRA.00096-19

**Published:** 2019-03-21

**Authors:** Yutian Feng, Artemis S. Louyakis, Andrea M. Makkay, Ray O. Guerrero, R. Thane Papke, J. Peter Gogarten

**Affiliations:** a Department of Molecular and Cell Biology, University of Connecticut, Storrs, Connecticut, USA; Georgia Institute of Technology

## Abstract

Isolated from Aran-Bidgol Lake in Iran, and reported here, Halorubrum ezzemoulense strain Fb21 represents the first complete genome from this archaeal species. Local recombination in this genome is in stark contrast to equidistant recombination events in bacteria. The genome’s GC bias, however, points to a genome architecture and origin that resemble those of a bacterium. Its availability, genome signatures, and frequent intragenomic recombination mean that Fb21 presents an attractive model organism for this species.

## Abstract (precis)

Isolated from Aran-Bidgol Lake in Iran, and reported here, Halorubrum ezzemoulense strain Fb21 represents the first complete genome from this archaeal species. Local recombination in this genome is in stark contrast to equidistant recombination events in bacteria.

## ANNOUNCEMENT

*Halorubrum ezzemoulense* is a globally distributed species (1) of the extremely halophilic archaea that commonly dominate alkaline lakes, salterns, and other hypersaline environments. *H. ezzemoulense* DSM 17463^T^ was isolated in 2006 (2) from Ezzemoul sabkha in Algeria. Despite numerous studies of this species and many high-quality draft genome sequence deposits in the NCBI database, to date there has been no complete genome sequence of *H. ezzemoulense*. Here, we present the first complete genome sequence of *H. ezzemoulense* strain Fb21, cultivated from hypersaline Aran-Bidgol Lake in Iran.

**FIG 1:**
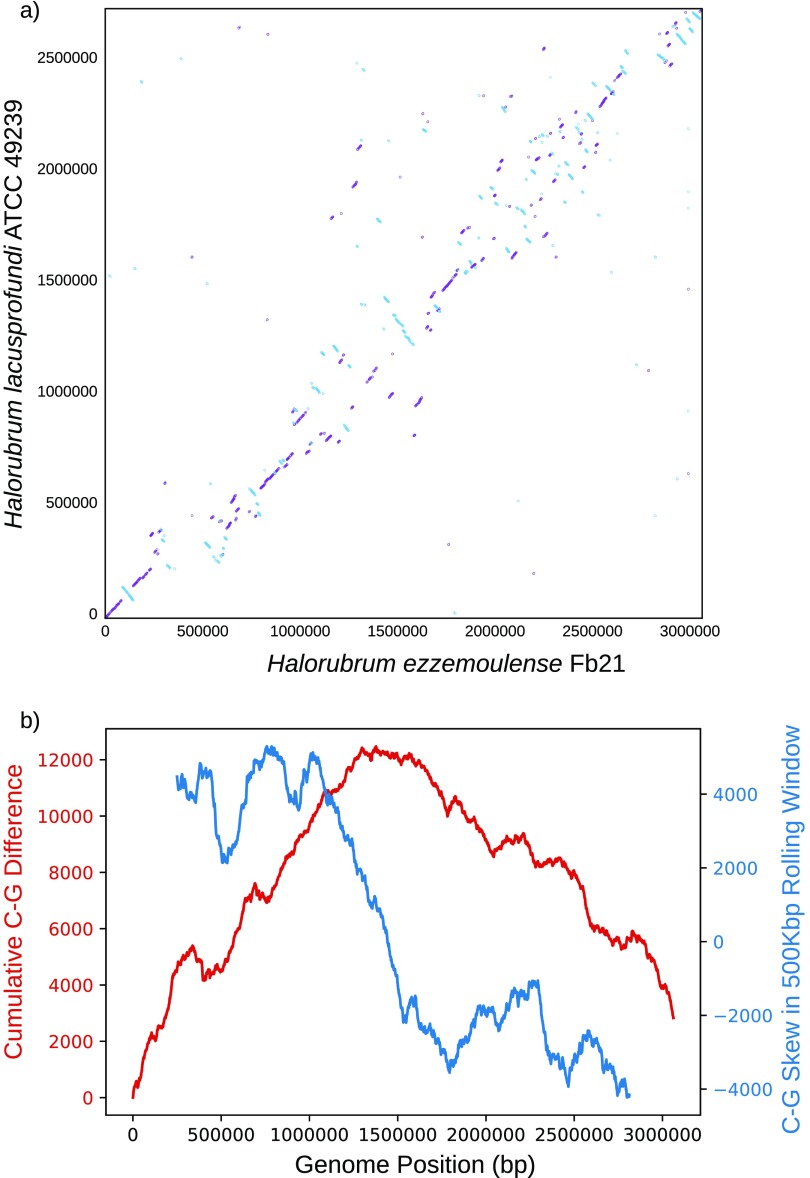
Analyses of the Halorubrum ezzemoulense Fb21 chromosome. (a) Nucmer comparison of *H. ezzemoulense* Fb21 (*x* axis) to *H. lacusprofundi* ATCC 9239 (*y* axis). Plus-strand best hits are shown in purple, while minus-strand best hits are shown in blue. Axis units are the chromosomal base positions. (b) The orange line represents cumulative C occurrences minus G occurrences (C − G) throughout the Fb21 chromosome, starting at the predicted main origin of replication. Values are calculated by subtracting the number of Gs encountered from the number of Cs at each chromosomal location. The blue line represents cumulative C − G strand bias in a 500,000-bp rolling window, calculated by the same method.

Fb21 was sampled in November 2007 from the shallow brine water column (10 cm, >200 practical salinity units) of Aran-Bidgol Lake (34°31′25′′ N; 51°53′40′′ E; 2,400 km^2^) and isolated as previously described (3). Briefly, DNA from Fb21 was isolated from pure liquid cultures and grown in Haloferax volcanii medium containing yeast extract, peptone, casamino acids, and 18% saltwater at 37°C (1, 4) using a phenol-chloroform-isoamyl alcohol (25:24:1) extraction followed by ethanol precipitation to purify the DNA, as described in the *Halohandbook* (5). Sequencing was completed using both short- and long-read platforms. Libraries were constructed using the Nextera XT DNA library preparation kit; two libraries were prepared for sequencing using the MiSeq reagent kit v2 (paired end, 500 cycles), a third used the MiSeq reagent kit v2 (paired end, 300 cycles), and each library was sequenced on separate runs on the MiSeq platform (Illumina, San Diego, CA) at the UConn MARS Center for Open Research Resources and Equipment. High-molecular-weight DNA was similarly isolated, with an additional cleanup using Agencourt AMPure beads (Beckman Coulter, Indianapolis, IN), and sequenced using a PacBio RS II single-molecule real-time (SMRT) DNA sequencing system on one SMRT cell long-read sequencing run on the Pacific Biosciences platform at the Keck Biotechnology Resource Laboratory at Yale University. All of the following programs were executed with default parameters unless otherwise stated. Reads were quality trimmed with Sickle v1.33 (6) and hybrid assembled with high-quality long reads using the Unicycler v0.4.7 pipeline (7) on the bold and normal settings. Contigs from the two assemblies were investigated and reconciled in Bandage v0.8.1 (8) to create the final assembly of the Fb21 genome. The assembly was then polished using the short reads with Pilon v1.130 (9). Three replicons were assembled and circularized, including a chromosome (∼3.1 Mbp, 68.46% GC content), a megaplasmid (∼606 kbp, 57.36% GC content), and a plasmid (∼57 kbp, 54.66% GC content). Total genome coverage with only the short reads was 99.94%, which was increased to 100% with the addition of long reads. The average sequencing depth across the genome with the short reads was 136× (standard deviation [SD] = 54.8), while the PacBio read average depth was 41× (SD = 17). Fb21 was previously determined to be a strain of *H. ezzemoulense* via 16S rRNA sequencing (1) and halobacterial lineage marker phylogenies in CheckM v1.0.12 (10). In addition, genome synteny between Fb21 and its closest neighbor, Halorubrum lacusprofundi ATCC 49239, was analyzed using nucmer v3.0 (11) and plotted (Fig. 1a). Numerous recombination events are visible in the plot. None of the detected recombination events are equidistant to the inferred origin of replication, unlike those found in bacterial genomes (12).

Archaeal mode in Prokka v1.13 (13) and the NCBI Prokaryotic Genome Annotation Pipeline (14) were used to predict and annotate the coding sequences (CDS) in the complete genome of Fb21. Of the 3,661 CDS identified, 3,443 were predicted to be proteins and 78 were predicted tRNAs, rRNAs, and noncoding (ncRNAs), while the remaining 218 were identified as pseudogenes. Aragorn v1.2.38 (15) and tRNAscan-SE v2.0 (16) were used to annotate 70 tRNAs, 6 rRNAs (2 identical operons), and 2 ncRNAs, all of which are on the chromosome. Using the *orc1*/*cdc6* gene (17) and read mapping, we predicted seven potential origins of replication on the chromosome, three on the megaplasmid, and one on the plasmid. The most likely origin of replication determined by similarity and GC strand bias (Fig. 1b) was rotated to the beginning of the chromosome. The cumulative and rolling window GC signatures indicate a bacterium-like genome architecture, which is unique to date among the Halobacteria.

We found that the chromosome of Fb21 contains a host of genes related to quorum sensing, rhodopsins, and chemotaxis, in addition to all necessary housekeeping genes. The megaplasmid contains two pseudogenes and two genes encoding restriction endonucleases, over fifty transposases, and an additional ribonucleotide reductase homolog. The genome shows evidence of local recombination events that are atypical of bacterial genomes. However, the GC strand bias profile of this archaeon resembles that of a bacterial genome. The Fb21 complete genome, in combination with the many other *H. ezzemoulense* draft genomes, makes this strain an excellent target for studies of within-species and within-population gene flow.

### Data availability.

The complete genome sequences and reports have been deposited in GenBank under accession numbers CP034940, CP034941, and CP034942 within BioProject accession number PRJNA513349 (assembly accession number GCF_004126515). The short and long reads are also available under the same BioProject accession number.
